# Condylar volume and condylar area in class I, class II and class III young adult subjects

**DOI:** 10.1186/1746-160X-8-34

**Published:** 2012-12-14

**Authors:** Matteo Saccucci, Michele D’Attilio, Daria Rodolfino, Felice Festa, Antonella Polimeni, Simona Tecco

**Affiliations:** 1 Department of Oral Science, Sapienza University of Rome, Rome, Italy; 2 Department of Medical, Oral and Biotechnological Sciences, University G. D’Annunzio, Chieti/Pescara, Italy

**Keywords:** Mandibular condyle, Volume, Class I, class II and class III, CBCT

## Abstract

**Aim:**

Aim of this study was to compare the volume and the shape of mandibular condyles in a Caucasian young adult population, with different skeletal pattern.

**Material and methods:**

200 Caucasian patients (15–30 years old, 95 male and 105 females) were classified in three groups on the base of ANB angle: skeletal class I (65 patients), skeletal class II (70 patients) and skeletal class III (65 patients). Left and right TMJs of each subject were evaluated independently with CBCT (Iluma). TMJ evaluation included: condylar volume; condylar area; morphological index (MI). Condylar volumes were calculated by using the Mimics software. The condylar volume, the area and the morphological index (MI) were compared among the three groups, by using non-parametric tests.

**Results:**

The Kruskal-Wallis test and the Mann Whitney test revealed that: no significant difference was observed in the whole sample between the right and the left condylar volume; subjects in skeletal class III showed a significantly higher condylar volume, respect to class I and class II subjects (p < 0.05); significantly lower condylar volume was observed in class II subjects, respect to class I and class III (p < 0.05). In the whole sample condylar volume (699.8 ± 63.07 mm^3^ in males and 663.5 ± 81.3 mm^3^ in females; *p* < 0.01) as well as condylar surface (423.24 ± 63.03 mm^2^ in males and 389.76 ± 61.15 mm^2^ in females; *p* < 0.01) were significantly higher in males than in females.

**Conclusion:**

Skeletal class appeared to be associated to the mandibular condylar volume and to the mandibular condylar area in the Caucasian orthodontic population.

## Introduction

Due to the role of the mandibular condyle in the development of the cranio-facial complex, evaluation of the condylar volume is one of the most debated arguments to improve knowledge about cranio-facial development. Since the mandibular condyle undergoes a remodelling process as it responds to continuous stimuli from childhood to adulthood, it is an important site of growth in the mandible, where its final dimension of shape and volume could be linked to the relation between the maxillary and mandibular bases [1-3].

Even in adulthood, the mandibular condyle seems to answer to functional demands, as its shape is continuously subjected to a remodelling process, which could affect its volume and shape [4-6]. As part of the temporo-mandibular joint (TMJ) structure, the mandibular condyle is considered to play a key role in the stability of long-term treatment results after orthodontic and orthognatic treatments .

In the orthodontic clinic, the mandibular condyle and the temporo-mandibular joint (TMJ) has been typically analyzed through the 2-D images. The recent advent of 3-D technology, in particular the cone beam computed tomography (CBCT) engineering, has overcome traditional CT scanners [7] and permits us a more complete analysis of the TMJ and the mandibular condyle than before. The CBCT produces images with isotropic sub-millimeter spatial resolution, and with a higher diagnostic quality providing a 3-dimensional representation of the maxillofacial hard tissues with minimal distortion [8].

In addition, it provides shorter scanning times of about 10–30 seconds, and radiation dosages of up to 15 times lower than those of conventional CT scans.

Condylar evaluations were previously made using 2-D images, combining axial sections with sagittal and coronal sections, or combining different radiographic techniques, in order to obtain an accurate measurement [9]. But 3-D technology has overcome the need of a costly and complicated combination of views or techniques. It reproduces multiple images on the axial, coronal and sagittal planes, with the possibility of viewing the images interactively and enhancing, consequently, the capability to identify the correct anatomy and the presence or absence of pathology.

It must be noted, however, that among typical CBCT systems, the Iluma is a particularly high dose system that is unsuitable for routine use in a young orthodontic population without careful professional judgement of imaging needs. For this, to familiarize with the cranio-facial complex, as seen in CBCT 3D reconstructions, new studies focusing on 3D images of the cranio-facial complex are needed.

Therefore, we can begin to identify a correlation between the cranio-facial morphology and condylar shape and volume with the new 3-D technologies such as the CBCT, which greatly enhances the analysis of cranio-facial development by identifying the shape of the condyle, and, more specifically, evaluating - with a higher rate of accuracy about the exact location and size - condylar linear measurements [10]. This correlation could eventually be a useful tool in improving clinical diagnosis and outcomes.

Thus, the aim of this study is to analyze the mandibular condyle volume, area and morphological index in young adult subjects without TMD dysfunction, evaluated with CBCT, in class I, II and III, and to evaluate whether the condylar volume and area can be related to skeletal class.

## Material and methods

### The sample

The 3-dimensional scans of 200 young adult Caucasian patients (15–30 years old, 95 males and 105 females), referring the Private study of radiology for orthodontic problems, were retrospectively analysed and retrieved from the computer data base. The sample was clinically evaluated to exclude the presence of signs and symptoms of temporomandibular disorders. The lateral films of the patients heads were extracted from the CBCT images and the Stainer cephalometric analysis was performed. The patient sample consisted of three groups, classified on the base of ANB angle: skeletal class I (65 patients), skeletal class II (70 patients) and skeletal class III (65 patients). All subjects gave their signed informed consent to the medical diagnostic procedure and to the use of data in this research. The University Ethics Committee approved the study, after careful consideration of its retrospective structure, and evaluation of medical records from the private radiological clinic.

Left and right TMJs were evaluated independently for each patient. TMJ evaluation included:

1. Condylar volume calculated with the Mimics software;

2. Condylar area, as surface measurements;

3. Morphological index, indicated as a ratio between surface and volume, constructed to reduce the differences among genders and subjects of different age, and to obtain a normalization of data.

### The volume calculation

Cone Beam Volumetric Tomography datasets were acquired with the ILUMA™ (IMTEC, 3 M Company, Ardmore, Oklahoma, USA), with a reconstructed layer thickness of 0.5 mm, with a 512x512 matrix. The device was operated at 120 kVp and 3–8 mA by using a high frequency generator with a fixed anode and a 0.5 mm focal spot. A single 40- second high-resolution scan was made of each skull. The voxel size was set at 0.25. Considering the high dose system of Iluma, for this protocol, the professional judgement of imaging needs was performed by an oral radiologist, after a clinical prescription by the individual dentist for each patient.

**Figure 1:**
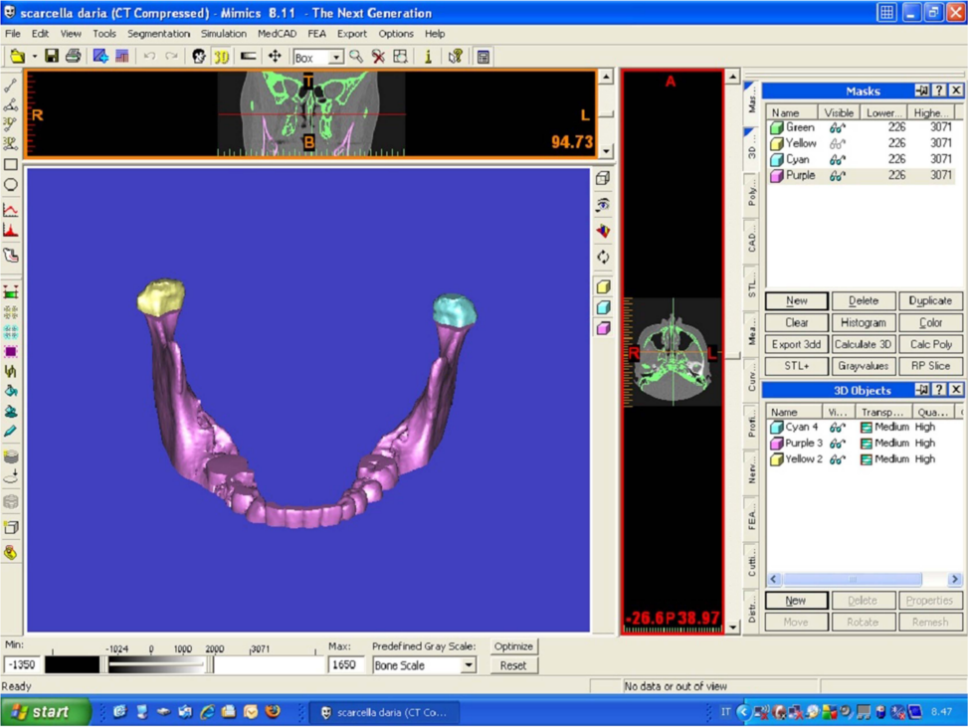
Mimics mask of a mandible.

The segmentation of the mandibular condyle was based on 2D Digital Imaging and Communications in Medicine (DICOM), created with CT data set, using the software Mimics™ 9.0 (Materialise NV Technologielaan, Leuven, Belgium) (Figure 1).

Each condyle was visualized in the recommended bone density range (range of gray scale from −1350 to 1650) isolated prior to making 3D measurements. Frankfort horizontal (FH) plane was constructed by creating a plane from the inferior orbital rim to the superior border of the external auditory meatus. An initial cut was made parallel to the FH plane just above the superior aspect of the condyle [11].

**Figure 2:**
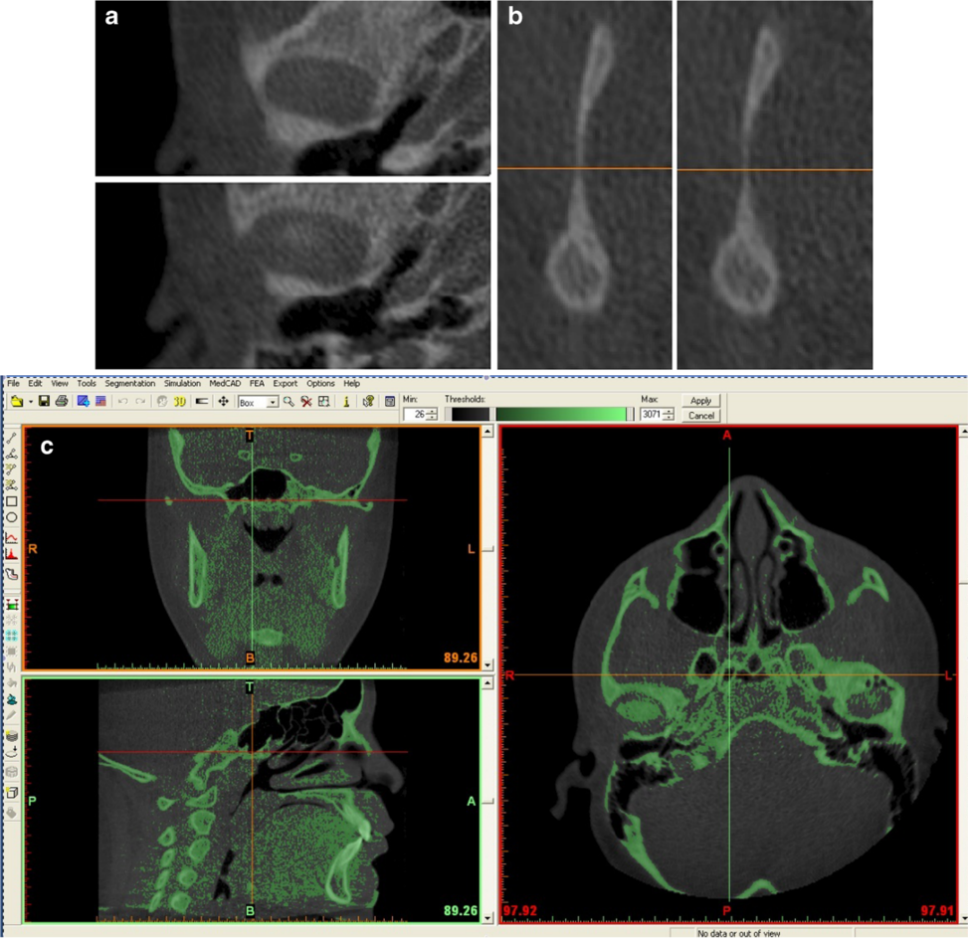
**(a) upper limit of condyle**; **(b) lower limit of condyle**; **(c) the mask obtained with the Mimics software.**

Then, the area of TMJ was enlarged, and the remaining surrounding structures were progressively removed using various sculpting tools for the upper, the lower and the side condylar walls, as showed in Figure 2a–c. The cuts were made on the coronal views; the upper, the lower and the side limits of the condyle were standardized.

**Figure 3:**
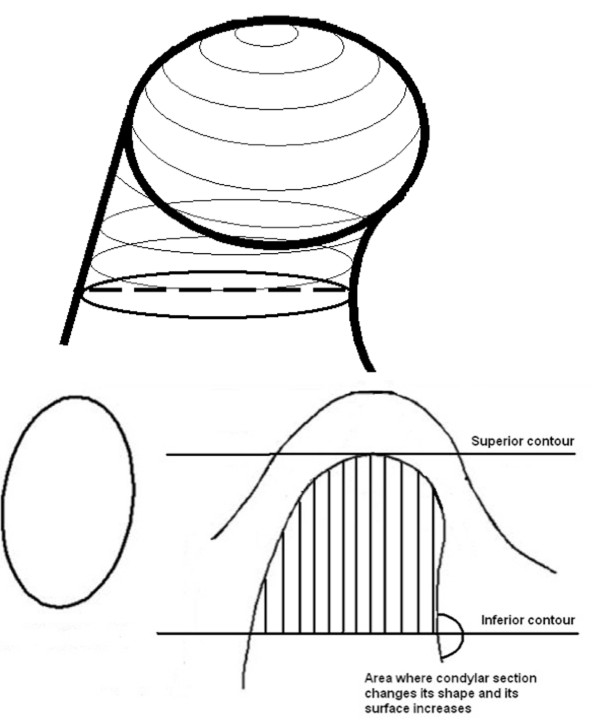
**(a) a scheme of condyle**; **(b) upper and lower limits of condyle.**

The difficulty of defining the exact contours of condyle was overcome by considering the density of cortical bone for the side walls of the condyle (Figure 2a–c). The upper limit of the condyle was defined where the first radiopaque area was viewed in the area of synovia (Figure 2a); then, for the lower sections, for each section, the condyle was isolated through the visualization of cortical bone. The lower limit of condyle was traced when the section left the elissoidal shape (due to the presence of the anterior crest) and become circular (suggesting the level of the condylar neck) (Figure 2b). The scheme of the limits is reported in Figure 3 a–b. Accordingly, the condyle CT data set were segmented with a dedicated Mimics™ tool to construct a mask, which included only the mandibular condyle (Figure 2c). After the isolation, three-dimensional multiplanar reconstructions were performed for each condyle using a Mimics tool (Figure 1). Volumetric measurements were made for each condyle with the Mimics™ automatic function.

### Studies on method error

To assess the intra-operator and inter-operator errors, due to the identification of condylar structure, the CBCT data of 10 patients were processed by the same operator (M.S) twice (with a gap of 1 week) and the differences in the condylar volumes and condylar areas were evaluated as method errors, then compared with the natural variance of the whole sample. No significant difference was reported between the two measurements of the volume (*Z* = −0.770; *p* = 0.441) or the area (*Z* = −1.784; *p* = 0.074).

The mean difference between the first and second measurements, and the relative contribution of errors to the total observed variations was determined for the two variables. The error variance (*Ve*) was calculated using the following formula:

$$\mathrm{Ve}=\sum {\left(x1-x2\right)}^{2}/2N$$

where *x*1 and *x*2 represent the first and second measurements, respectively, and *N* is the sample size. Accordingly, for the volume, the error variance was 3.77 and for the area it was 3.44.

The mean differences between the first and the second measurements were 1.42 mm^2^ and 0.85 mm^3^. In general, the contributions of intra-operator method errors to the total variance were found to be relatively insignificant: 0.06% for the volume and 0.08% for the area.

Subsequently, to assess the inter-operator method error, the CBCT data of 10 subjects were also processed by another researcher (M.D) and the data compared using Mann–Whitney U test. Mann–Whitney U value is 48.00 for volume and 49.00 for surface, with no significant difference.

For the inter-observer method error, the variance error was 4.34 for the volume and 4.43 for the area.

The mean differences between the first and the second operators were 0.61 mm^2^ and −1.71 mm^3^. In general, the contributions of inter-operator errors to the total variance were found to be relatively insignificant: 0.08% for the volume and 0.1% for the surface.

### Data analysis

Data were analyzed using SPSS 14.0 (SPSS Inc, Rainbow Technologies, Chicago, Ill). Significance testing for differences in volumetric and surface measurements among the three groups was accomplished using Kruskal-Wallis H test and Mann–Whitney U test. The *p* value was set at 0.05.

## Results

### Condylar volume and area

For 3D measurements, significant differences were found between the measurements obtained for the class III group, which showed a significant higher volume and area, than class II subjects (p < 0.05). Significantly lower condylar volume was observed in class II subjects, respect to class I and class III (p < 0.05). (Table 1 and Table 2). Table 3 reports the data about the MI.

**Table 1:** **Descriptive statistic for the variable Volume** (**mm**^**3**^) **calculated in the three groups**

	**N**	**Mean**	**Std**. **Deviation**	**Range**	**Minimum**	**Maximum**	**Kurtosis**	
							**Statistic**	**Std**. **Error**
CLASS II subjects								
age	68	19.20	4.27	18.00	12.00	30.00	−.611	.574
Volume (right)	68	2350.64 *	642.77	2743.34	1032.34	3775.68	−.128	.574
Volume (left)	68	2352.02 *	733.33	3920.44	832.76	4153.20	.482	.574
CLASS I subjects								
age	65	20.86	7.50	18.00	12.00	30.00	1.785	.586
Volume (right)	65	2693.09	538.48	1761.95	1637.45	3399.40	−.213	.586
Volume (left)	65	2675.09	444.93	1319.31	2040.97	3360.28	−1.114	.586
CLASS III subjects								
age	65	17.7385	6.8949	19.00	10.00	29.00	−1.195	.586
Volume (right)	65	2672.80 *	599.66	1713.00	2039.23	3752.23	−.890	.586
Volume (left)	65	2792.78 *	648.29	1923.01	1816.34	3739.35	−1.170	.586

For right volume: Chi-square: 10.367; p = 0.006 (Kruskal-Wallis test with skeletal class as grouping variable); Mann-Whitney U = 1651.00; p = 0.012 (post-hoc evaluation);

For left volume: Chi-square: 11.814; p = 0.003 (Kruskal-Wallis test with skeletal class as grouping variable); Mann-Whitney U = 1612.00; p = 0.007 (post-hoc evaluation).

**Table 2:** **Descriptive statistic for the variable Surface** (**mm**^**2**^) **calculated in the three groups**

	**N**	**Mean** (**mm**^**2**^)	**Std**. **Deviation**	**Range**	**Minimum**	**Maximum**	**Kurtosis**	
							**Statistic**	**Std**. **Error**
CLASS II subjects								
Surface (right)	68	**1145**.68*	197.67	908.66	729.21	1637.87	.568	.574
Surface (left)	68	**1185**.52*	197.26	1037.24	765.70	1802.94	1.083	.574
CLASS I subjects								
Surface (right)	65	**1210**.**50**	191.92	672.56	881.18	1553.74	--.186	.586
Surface (left)	65	**1226**.**49**	187.09	595.04	1039.68	1634.72	.646	.586
CLASS III subjects								
Surface (right)	65	**1365**.76*	226.74	673.54	1084.63	1758.17	−.933	.586
Surface (left)	65	**1362**.55*	263.18	670.74	1055.72	1726.46	−1.523	.586

For right surface: Chi-square: 10.367; p = 0.006 (Kruskal-Wallis test with skeletal class as grouping variable); Mann-Whitney U = 1108.00; p = 0.001 (post-hoc evaluation);

For left surface: Chi-square: 12.104; p = 0.002 (Kruskal-Wallis test with skeletal class as grouping variable); Mann-Whitney U = 1603.00; p = 0.006 (post-hoc evaluation).

**Table 3:** **Descriptive Statistics for the variable** “**Morphological Index**” (**volume**/**surface**)

	**Range**	**Minimum**	**Maximum**	**Mean**	**Std**. **Deviation**	**Variance**	**Kurtosis**	
	**Statistic**	**Statistic**	**Statistic**	**Statistic**	**Statistic**	**Statistic**	**Statistic**	**Std**. **Error**
CLASS II subjects								
Morphological Index (right)	1.18	1.35	2.53	2.01	.29	8.445E-02	−. 271	.574
Morphological Index (left)	2.45	.19	2.64	1.95	.42	.180	3.729	.574
CLASS I subjects								
Morphological Index (right)	.68	1.86	2.54	2.21 *	.19	3.762E-02	−.186	.586
Morphological Index (left)	.51	1.96	2.47	2.17 *	.14	2.152E-02	.081	.586
CLASS III subjects								
Morphological Index (right)	.45	1.68	2.13	1.94 *	.18	3.330E-02	−1.521	.586
Morphological Index (left)	.48	1.72	2.20	2.03 *	.16	2.588E-02	−. 262	.586

Valid N (listwise).

For the right MI: Chi-square = 46.819; p = 0.000 (Kruskal Wallis test with skeletal class as grouping variable); Mann-Whitney U = 585.00; p = 0.000 (for the post-hoc analysis).

For the left MI: Chi-square = 30.226; p = 0.000 (Kruskal Wallis test with skeletal class as grouping variable); Mann-Whitney = 1008.00; p = 0.000.

## Discussion

### Age related differences

In this study, we only included the data of young adult subjects (from 15 to 30 years old); this was done because older subjects are expected to have more frequent and severe progressive degenerative conditions due to the development of TMJ osteoarthritis (such as flattening, erosion, sclerosis, osteophytes, resorption, which can affect the condylar volume and its position in the fossae) than younger patients. We did not perform any statistical comparison between older and younger subjects because only a few subjects were near 15 years of age. The use of a normalized variable such as the morphological index reduced the error associated to differences among subjects of different age [12].

### Sexual dimorphism

The condylar surface area was significantly higher in males than in females (*p* < 0.001), as well as condylar volume (*p* < 0.01).

The differences between the mean percentages of males and females are in accordance with those of a recent study that investigated the female-to-male proportions in head and facial linear dimensions, and we found a mean difference of 3–5% in the frontal and lateral views in young and adult patients, between males and females [13].

The wide range of values and standard deviations in volume or surface suggests high variability among the subjects. But, this evidence has any clinical relevance or role in the TMD, considering that no subject included in this report had any signs or symptoms of TMDs.

For the variable MI that indicates the ratio between volume and surface, the difference between females and males is about 2.8% of the MI in the whole sample.

### Differences related to skeletal class

In this study, we observed a greater volume of the condyle with the subjects in skeletal class III, respect to subjects in skeletal class II and skeletal class I. A previous study has demonstrated that hyperplasia of the mandibular condyle is characterized histologically by the presence of an uninterrupted layer of undiffentiated germinative mesenchyme cells, a layer of hypertrophic cartilage and the presence of islands of chondrocytes in the subchondral trabecular bone.

Thus, it could be interesting compare our data with histology, in order to investigate whether the different volume observed by us corresponds to different histological aspects of cartilage. In a recent study [14], including 15 patients with severe skeletal Class II (mean age 18.0 yrs) and 14 patients with severe skeletal Class III (mean age 19,2 yrs), undergoing a combined orthodontic and orthognathic treatment, CT examination was performed, and height and width of condyle, height of procesus condylaris measured in two dimension projection (2D). There were statistically significant differences between two study groups for all spatial measurements on both sides with larger spatial measurements in patients with Class II malocclusions. Our results are not in agreement with this as we observed a smaller condylar volume and area in class II subjects. The difference was probably due to the different severity of malocclusion in the two samples.

It is known that there are differences in the force vector against the condyle during mastication in the different subjects, as assessed previously [15]. The direction of the force vector of the class II subjects appears significantly larger than those of the class I and III. Skeletal class III malocclusions in Japanese adolescents tend to show an asymmetry of the condylar inclination when compared with those of class I and class II malocclusion, studying a Sectograph [16].

There are a few reports that TMJ morphology has a strong correlation with skeletal morphology [17] and exclusively an inverse relationship between the angle of the articular eminence and the occlusal and the mandibular planes [18]. Skeletal class III pattern tended to be more closely associated with the asymmetry of condylar inclination than skeletal I and II groups [19-21]. In the scientific literature, the condylar volume has been also related to the type of mastication . Twenty-five 3-week-old (at the time of weaning) imprinting control region mice were randomly divided into three groups: mice fed a hard diet, mice fed a soft diet, and mice alternately fed hard and soft diets every week for 4 weeks. The condylar width was significantly greater in the hard diet group than in the soft diet group after 1 week. Bone volume (of the whole mandible) resulted significantly less in the soft diet group than in the other two groups after 4 weeks. These findings suggest that changes in mastication markedly affect mandibular condylar cartilage growth and mandibular morphology, as well as the skeletal class.

According to other studies, the articular cartilage – a relevant site of growth – has been demonstrated to respond to the degenerative changes and nonphysiological strain in the joint areas (application of soft diet or extractions), through changes in the thicknesses of single cartilage layers and total layer thickness, causing a change in the vertical dimensions and width, which is manifested by changes in the maturation processes of centrally unloaded cartilage sections in rats (6).

From a clinical point of view, the functional loads applied to the TMJ might influence TMJ’s morphology; the shape and function are intimately related, although this concept is given due importance only in studies on class II and class III skeletal patterns , both the volume and the area of a condyle differ between the genders and the subjects with different skeletal class [22].

With the advent of 3-D CBCT scan, the clinician can request the radiologist to directly evaluate or calculate condylar volume and area, as also the MI, using dedicated software. It was shown that the ratio of bone surface to volume correlates with the degree of bone mineralization and the number of condylar trabeculae in a model of porcine mandibular condyle, indicating a correlation of this variable with the data demonstrating the modeling or remodeling of the bone [23].

### Limits of the study

Numerous factors should be considered in applying the results of this investigation to clinical situations. The 3-D volumetric depiction depends on the appropriateness of segmentation, the threshold of bone voxel values, and the accurate suppression of the surrounding tissue values to enhance the structure of interest. The depiction is dependent on the software algorithm, the spatial and contrast resolution of the scan, the thickness and degree of calcification or cortication of bone structure, and the technical skill of the operator. The Mimics software used in this study enables semi-manual segmentation by interaction of the operator with the data to produce a visually acceptable 3-D rendering. According to Periago [24], these limitations cause deficiencies or voids in the surface of the image, which occur in regions represented by few voxels or that have gray values still representing the bone, but outside the threshold. These areas include the cortical bone of the mandibular condyle, and thus may lead to greater identification error (e.g., for condylar contours) and consequently to measurement error. However, no significant difference was found between the intra- and inter-observer method errors, thus suggesting that accurate procedure of segmentation could restore itself from errors.

Finally, this study was restricted to Caucasian patients. Future studies will be directed to evaluate ethnic and racial differences.

## Conclusion

In the present study, using the CBCT-based method, we demonstrated that condylar volume and area can be related to skeletal class in the Caucasian orthodontic population.

### Clinical relevance

Furthermore, the generation of stable and repeatable data on condylar volume and area in functionally normal joints will form the basis for future studies on cranio-facial development, and the measurements of condylar volume and area, and their relation with the cranio-facial complex.

## Competing interests

The authors declare that they have no competing interests.

## Authors’ contributions

MS is the Lead author of this research article. He 1) has made substantial contributions to conception and design of the manuscript, 2) have been involved in drafting the manuscript or revising it critically for important intellectual content; 3) has given final approval of the version to be published. ST is the Principal investigator of this research article. She 1) has made substantial contributions in drafting and in conception of the manuscript, 2) acquisition, analysis and interpretation of data. AP, DA, DR and FF participated in drafting the manuscript and helped in the revision of the manuscript. All authors read and approved the final manuscript.
